# Spatial risk mapping of highly pathogenic avian influenza in Morocco using geographic information system and multi-criteria decision analysis: Implications for targeted surveillance and control

**DOI:** 10.14202/vetworld.2025.3713-3730

**Published:** 2025-12-07

**Authors:** Fadoua Boudouma, Hicham Hajji, Mariette Ducatez, Oumayma Arbani, Kenza Aitelkadi, Siham Fellahi

**Affiliations:** 1 Department of Animal Health Regulation, National Office of Food Safety, 10000 Rabat, Morocco; 2 Avian Pathology Unit, Agronomy and veterinary Institute, Hassan II, BP 6202, Rabat, Morocco; 3 Department of Cartography-Photogrammetry, Agronomy and Veterinary Institute, Hassan II, BP 6202, Rabat, Morocco; 4 Host-Pathogen Interactions Unit (IHAP), Université de Toulouse, INRAE, ENVT, 31300 Toulouse, France

**Keywords:** avian influenza, biosecurity, geographic information system, Morocco, multi-criteria decision analysis, risk mapping, surveillance

## Abstract

**Background and Aim:**

Highly pathogenic avian influenza (HPAI) remains a global threat to poultry production, trade, and public health. While Morocco has not yet reported confirmed HPAI outbreaks, the endemic circulation of low-pathogenic avian influenza (LPAI) H9N2 since 2016, proximity to affected neighboring countries, and Morocco’s position along migratory bird flyways highlight the country’s vulnerability. This study aimed to identify high-risk areas for HPAI introduction and spread to inform risk-based surveillance and control policies.

**Materials and Methods:**

We applied a spatial multi-criteria decision analysis integrated with geographic information systems at the provincial scale. Relevant risk factors were identified through a literature review and expert consultation, and categorized into the introduction (wetlands, live poultry imports, recreational bird imports, and poultry products) and spread (poultry density and type, live bird markets, transport networks, and human population density) domains. Weights were assigned to factors using the analytic hierarchy process based on responses from 73 poultry-sector experts. Data were normalized, integrated into composite risk maps, and validated against historical LPAI H9N2 outbreak data (2016). Sensitivity and uncertainty analyses were used to assess model robustness.

**Results:**

The final maps revealed that 25 provinces (33.3% of the national territory) exhibited high-to-very high risk of HPAI introduction, particularly along northern coastal provinces, border regions, and areas linked to recreational bird imports. For spread risk, 41 provinces (41.3%) were classified as high to very high, concentrated in the Casablanca–Settat, Rabat–Salé–Kenitra, Fès–Meknès, and Marrakech–Safi regions, which are characterized by dense poultry production, major trade hubs, and extensive transport networks. Sensitivity analyses confirmed the model’s stability, with variations in weight producing a minimal impact on risk classifications.

**Conclusion:**

This study provides the first comprehensive spatial risk maps of HPAI introduction and spread in Morocco, highlighting priority provinces for early detection, targeted surveillance, and preventive biosecurity measures. Despite limitations arising from reliance on LPAI data and expert judgment, the approach offers a robust decision-support tool for veterinary authorities. The methodology is adaptable to regional applications and can be refined with real-time surveillance data, enhancing Morocco’s preparedness and resilience against future avian influenza incursions.

## INTRODUCTION

Avian influenza is a highly contagious viral disease with a pronounced capacity for rapid dissemination. It is associated with substantial mortality in both domestic and wild bird populations, resulting in severe economic losses in poultry production and representing a potential zoonotic threat to human health. In recent years, highly pathogenic avian influenza (HPAI) outbreaks have been documented across Africa, Asia, and Europe [1]. Given its consequences for poultry farmers’ livelihoods, international trade, and public health, the establishment of robust surveillance systems for wild birds and poultry is of paramount importance.

The circulation of HPAI subtypes H5N1 and H5N8 in Morocco’s neighboring countries, including Algeria [2], Mauritania [3], Senegal [4], and Egypt [5], as well as in several European nations [6], poses a serious transboundary risk. This threat is further magnified by the presence of migratory bird flyways [7] and the persistence of informal poultry trade networks [8, 9], highlighting the urgent need to investigate the local determinants of HPAI introduction and spread in Morocco.

Until December 2015, Morocco had not reported any cases of avian influenza. However, in January 2016, outbreaks occurred on several broiler farms, characterized by respiratory distress, reduced feed intake, and elevated mortality. Laboratory investigations confirmed low pathogenic avian influenza (LPAI) subtype H9N2 as the causative agent [10]. Subsequent epidemiological monitoring and molecular studies by El Mellouli *et al*. [11] and Arbani *et al*. [12] have demonstrated the continued endemic circulation of LPAI H9N2 in Moroccan poultry populations.

Globally, numerous studies have sought to map the risk of HPAI emergence. These efforts typically employ geospatial statistical methods to identify natural and anthropogenic drivers [13] and integrate geographic information systems (GIS) with multi-criteria decision analysis (MCDA) to assess the determinants of viral introduction and spread [14]. Such research emphasizes the need to understand epidemiological drivers to design risk-based surveillance and control strategies [15].

Nevertheless, incomplete epidemiological and statistical datasets frequently constrain mapping initiatives. To address these limitations, knowledge-based modeling approaches, such as spatial MCDA and the multi-criteria hierarchy method, have been applied to produce predictive maps in regions free of reported outbreaks [16, 17]. The spatial MCDA framework relies on decision rules derived from prior knowledge to delineate areas most vulnerable to disease introduction and dissemination [18], operating under the assumption of spatial stability between explanatory variables and epidemiological outcomes [19].

The principal steps of MCDA include identifying relevant risk factors, defining their relationship to disease occurrence, assigning weights, integrating the weighted factors into risk maps, and conducting sensitivity and validation analyses [20]. The first three steps are generally informed by literature review and expert consultation. A pivotal component of the MCDA approach lies in the accurate identification of introduction and spread risk factors, which have been consistently highlighted in the scientific literature [21–24].

Despite the extensive body of global research on avian influenza risk mapping, most existing studies have focused on countries with recurrent outbreaks where epidemiological and surveillance data are readily available. Morocco, however, presents a unique situation. While LPAI H9N2 has been endemic since 2016, no confirmed HPAI outbreaks have been reported to date. This absence of outbreak data has limited the development of predictive epidemiological models specifically tailored to the Moroccan context. Moreover, transboundary threats from neighboring countries and the influence of migratory bird flyways remain poorly quantified. Current national surveillance strategies rely primarily on passive and active detection systems rather than on spatially explicit, evidence-based scientific risk assessment approaches that integrate geospatial modelling, epidemiological data, and quantifiable risk factors. As a result, there is limited understanding of how environmental, epidemiological, and anthropogenic factors, such as poultry density, live bird trade networks, transport infrastructure, and wetlands frequented by migratory species, interact to shape Morocco’s vulnerability to HPAI introduction and spread. Addressing this gap is essential for establishing proactive, risk-based surveillance and control strategies.

This study aims to develop the first comprehensive spatial risk maps of HPAI introduction and spread in Morocco by integrating MCDA with GIS. By systematically identifying, weighting, and combining risk factors relevant to the Moroccan poultry sector and environment, the study seeks to pinpoint provinces most at risk of HPAI incursion and subsequent dissemination. Expert input from poultry veterinarians and stakeholders was incorporated to ensure contextual relevance, while sensitivity and uncertainty analyses were conducted to validate model robustness. The resulting risk maps are designed to serve as practical decision-support tools for veterinary authorities and policymakers, guiding the prioritization of surveillance efforts, allocation of resources, and implementation of targeted preventive measures. Beyond Morocco, the methodology offers a transferable framework that can be adapted to other countries with limited outbreak data but significant exposure to avian influenza risks.

## MATERIALS AND METHODS

### Ethical approval

The study did not involve any experimental manipulation or direct animal handling. All data used were obtained from secondary sources, including publicly available epidemiological databases, official veterinary records, and expert surveys. Expert participation in the questionnaire survey was entirely voluntary, and informed consent was obtained from all participants prior to data collection. The survey was conducted in accordance with the ethical standards of the Institut Agronomique et Vétérinaire Hassan II (Rabat, Morocco), following national guidelines for research involving human participants (Reference No. IAVH2/DAH/2022/045). The study protocol and data management procedures were reviewed and approved by the Institutional Ethics Committee of the Institut Agronomique et Vétérinaire Hassan II, ensuring compliance with Moroccan ethical regulations and the principles outlined in the Declaration of Helsinki for the use of non-clinical human data. No live animals were sampled or subjected to experimental procedures during this work.

### Study period and location

The study was conducted from September 2022 to July 2024 and covered the entire territory of Morocco. All administrative provinces were included in the analysis to ensure nationwide representation. Data collection and spatial analyses were performed at the provincial level.

### MCDA method

The MCDA method is widely used to engage stakeholders, as it enables the inclusion and evaluation of conflicting criteria while integrating expert opinions to guide the risk prioritization process. Several studies have used multi-disciplinary and multi-domain experts to prioritize risks.

The key steps of our MCDA selection approach included factor selection, defining relationships between risk factors and disease, assigning weights to factors, combining risk factors to create risk maps, conducting sensitivity analysis, and validation. The initial stage, factor selection specific to the Moroccan context, environment, and policy, relationship definition, and weight assignment, was conducted through a literature review and expert consultations. However, validating maps produced by knowledge-based models remains challenging, particularly in epidemiological studies, where limited disease data often restricts validation to visual comparisons with existing data sources.

### Model validation

The predictive performance of the MCDA models was assessed using the receiver operating characteristic curve (ROC) analysis, with the area under the curve (AUC) as a quantitative metric. The predicted risk values were derived from the maps by calculating the average risk of each province. Observed values indicating the presence or absence of an LPAI outbreak were collected by the National Office of Food Safety (ONSSA) between January 28 and March 14, 2016. For validation purposes, outbreak data were split into two periods: The first 14 days of the epidemic were used to validate the risk map for virus introduction, whereas outbreaks occurring afterward were used to assess the risk map for virus propagation. ROC curves and AUC values, including confidence intervals, were calculated in R to evaluate the model’s discriminative ability and quantify uncertainty.

### Process workflow

The study was conducted over a period of 2022–2024, allowing for the collection, processing, and analysis of all necessary data. This timeframe allowed sufficient time to complete the preparation, data acquisition, geospatial processing, and results interpretation stages. The methodology followed in our work comprised five main phases, summarized in Graphic 1.

**Graphic 1:**
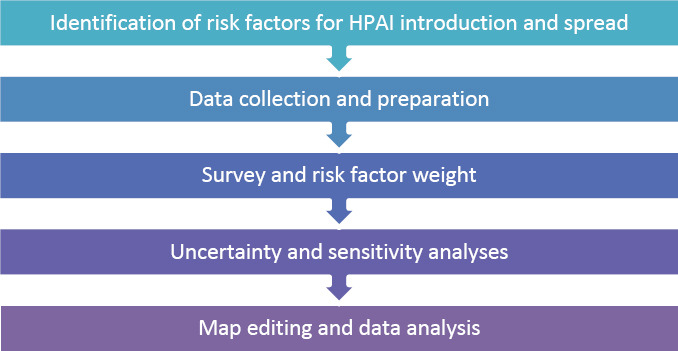
The general methodology of the study.

### Identification of risk factors

We began our study with a literature review to identify the risk factors for the introduction and spread of HPAI, as reported in previous studies [14, 15, 22, 23, 25]. We selected the factors most relevant to the Moroccan context and for which data could be reliably obtained from this list.

Tables 1 [9–11, 14, 25–32] and 2 [1, 11, 23, 33–40] summarize the risk factors of HPAI introduction and spread, respectively. We list the factors with their Moroccan context and source material.

**Table 1:** Risk factors for the introduction of HPAI.

Risk factors	Risk factor and Moroccan context	Data sources
Wetland sites	The spread of the HPAI virus is closely linked to the seasonal migration of aquatic birds [26, 27]. The geographical overlap of major migratory flyways creates a network that facilitates viral exchange between avian populations, particularly in wetland areas that serve as gathering points [27, 28]. Both symptomatic individuals and asymptomatic carriers can shed the virus, thereby promoting its dissemination over long distances. Transmission to domestic poultry occurs primarily in settings where interaction with wild birds is possible, particularly through contaminated water sources or direct contact [14, 28].	We used the official Moroccan database of sites of biological and ecological interest, most recently updated in 2023, to construct the wetland data layer, which includes 38 wetlands, supplemented with RAMSAR sites [29, 30]. Each wetland was delineated in QGIS through the following steps: first, the QuickOSM extension was used to extract data and generate polygon files. Second, the wetland layer was clipped to Morocco’s provincial administrative boundaries using the *clip* tool. Finally, the surface area of wetlands within each province was calculated.
Importation of live poultry and poultry products	The importation of live poultry and related products poses a high risk of avian influenza, especially when originating from infected areas. Even if asymptomatic, infected birds can shed the virus during transport, facilitating its spread [29]. Unprocessed poultry products may also be contaminated, particularly hatching eggs, which can transmit the virus to chicks upon hatching. Several studies have well-documented the role of both formal and informal trade in the introduction of avian influenza viruses [9, 25, 29].	In Morocco, the introduction of live poultry – primarily hatching eggs of breeder type – is mainly carried out through seaports and international airports located in the provinces of Casablanca and Tangier. The data, along with the types of products, were collected from the database of the ONSSA, published in 2023.
Importation of recreational birds	The introduction of recreational birds, particularly falcons, can significantly contribute to the introduction and spread of influenza viruses if they are not subjected to rigorous veterinary checks upon arrival [28, 30]. These birds are often imported for activities such as falconry, hunting, or exhibitions, and sometimes originate from regions where the virus is endemic [31]. The introduction of recreational birds, particularly falcons, into Morocco is primarily for hunting purposes [10, 11]. Morocco has a legal framework in place to control such importations, including the requirement to present a health certificate and implement sanitary inspections at border points. In Morocco, the ECWP has several breeding and sustainable management sites for the Houbara bustard, a falconry species. This activity aims to provide birds for falcon hunting, which is practiced in certain provinces of Morocco. The frequent importation of falcons from countries where HPAI strains are endemic exposes Morocco to an increased risk of introduction of the virus.	In our study, we targeted the provinces of Boulemane, Figuig, Midelt, and Tata, where falcon hunting is practiced. This practice is closely linked to the implementation of the ECWP, which involves releasing birds for hunting in these provinces [32].

ECWP = Emirates center for wildlife propagation, RAMSAR = Ramsar convention on wetlands, HPAI = Highly pathogenic avian influenza, ONSSA = National office of food safety.

**Table 2:** Risk factors for the spread of HPAI.

Risk factors	Risk factor and Moroccan context	Data sources
Density and type of poultry production	The density and type of poultry farms play a critical role in the spread of AIVs. The risk of virus transmission is significantly higher in areas where poultry farms are numerous and geographically close together [33, 34]. In Morocco, the implementation of biosecurity measures varies significantly across poultry production systems, creating differential risks for the spread of HPAI.	To illustrate this risk, three key factors were integrated: Poultry farm density, production type, and biosecurity levels. We consulted experts to quantify the level of biosecurity and assign a score for each province. Supplementary Table 1 presents the ONSSA database, which outlines the number and types of production across the provinces.
Live poultry and table eggs markets	The congregation of birds from diverse sources, combined with inadequate biosecurity measures and high animal density, creates an environment that is highly conducive to the amplification and spread of avian influenza viruses [1, 35]. These markets have been identified as key nodes in the epidemiological network of avian influenza, enabling rapid and often undetected spread to farms and slaughterhouses [33, 36]. In Morocco, half of the table eggs and at least three-quarters of the slaughtered poultry pass through wholesale markets.	Morocco has two main wholesale markets: Casablanca and Rabat.
Movement of means of transport linked to the poultry sector	The vehicles used, whether for delivering feed or transporting live birds and eggs, present a significant risk of spreading AIVs. This risk is exacerbated by inadequate application of biosecurity protocols, particularly the lack of systematic cleaning and disinfection of vehicles between operations. These vehicles can act as mechanical vectors, allowing the virus to travel considerable distances and contaminate previously unaffected farms, markets, and processing facilities [37, 38].	Given the complexity of collecting data on this factor, we used the density of the Moroccan road network by province, including motorways and first, second, and third category roads, as an indicator to map this factor. To represent this factor, we used the density of the Moroccan road network by province, including motorways and roads of the first, second, and third categories. The road data were obtained from open-source datasets (2022) and processed by extracting all roads with QuickOSM, calculating their total length per province, and dividing by the provincial area (km^2^) to obtain road density.
Density of the human population	Human population density has been identified as a major risk factor for the spread of HPAI [23, 33, 39]. As an indirect indicator of epidemiological processes, high population density reflects conditions that increase virus transmission through trade and agricultural practices. In Morocco, this factor is strongly linked to the presence of traditional poultry markets (souks), slaughterhouses, and egg-selling points. The existence of such sites amplifies the risk of HPAI virus circulation across areas with different population densities. This risk is further exacerbated by the absence or poor enforcement of biosecurity and hygiene measures, thereby significantly increasing poultry farms’ vulnerability to viral dissemination [11].	The population density was calculated by dividing the number of inhabitants per province (HCP, 2014 census data) by its surface area (km^2^) [40].

HPAI = Highly pathogenic avian influenza, AIV = Avian influenza virus, ONSSA = National office of food safety, HCP = High Commission for Planning.

### Data collection and typology

The spatial assessment of avian influenza introduction and spread risks requires the integration of semantic data with geographic components. Alphanumeric datasets were collected from local veterinary authorities and institutional reports. The typology of the data was categorized according to risk factor domains: Poultry production metrics (production type, farm density, and flock sizes), environmental variables (wetland locations and migratory bird corridors), and socioeconomic factors (live bird trade routes and market networks).

The study was conducted on a national scale, covering the entire territory of Morocco, including the 12 administrative regions and 69 provinces. All spatial analyses were performed using vector shapefiles, which are standard GIS data formats that store both the geographic boundaries and attribute information of spatial units. These shapefiles included polygon layers representing provincial limits and were used to spatially link datasets to their corresponding provinces. The province was the smallest spatial unit of analysis, which defined the resolution of the resulting maps.

The choice of provinces as the unit of analysis was guided by the availability and consistency of epidemiological and poultry sector data, as well as by the provincial-level organization of official surveillance and reporting activities in Morocco.

The coordinate reference system used was European Petroleum Survey Group (EPSG):4326, World Geodetic System (WGS) 84. The maps were generated from data collected during the study.

### Expert survey and weighting of risk factors

To identify the risk factors associated with the introduction and spread of HPAI in the Moroccan context, we relied on the expertise of veterinarians working across different poultry farming sectors. Their input was essential in identifying the key risk factors to be considered. For this purpose, a list of contacts was compiled, including practicing veterinarians, public-sector veterinarians (administrations and public establishments), researchers, and pharmaceutical industry professionals. The profiles of the experts who participated in the survey are provided in the Supplementary File.

An electronic questionnaire was designed using Google Forms. A preliminary version of the questionnaire was first reviewed by a selection of experts, whose feedback led to several refinements. The final validated version is provided in the Supplementary File.

The questionnaire was in the French language and divided into two main sections: The first dealing with risk factors for the introduction of HPAI and the second with risk factors for its spread. Each section contained pairs of risk factor combinations, and the expert had to choose from the proposed answers. After the questionnaire was sent out, 73 experts replied, and their opinions were translated into numerical values using the Saaty scale [41]. The Saaty scale is a nine-point numerical scale used in the Analytic Hierarchy Process (AHP) to compare pairs of criteria or alternatives according to their relative importance. It allows experts to express judgments ranging from equal importance (1) to extreme importance (9).

All experts who participated in the survey provided informed consent to the publication of the data.

To establish the relative weight of each identified risk factor, pairwise comparisons were conducted with the participating experts using the Analytic Hierarchy Process (AHP), which has been applied in numerous fields [42–44]. The AHP, developed by Saaty in the 1970s [41], is a widely used multi-criteria decision-making method. It enables decision-makers to rank alternatives when multiple, and sometimes conflicting, criteria are involved. The problem is decomposed into a hierarchy consisting of the main objective, criteria (and subcriteria, if applicable), and alternatives. Experts then perform pairwise comparisons between criteria using Saaty’s scale of 1 (equal importance) to 9 (extreme importance) to express their relative importance. These judgments are mathematically processed to derive the weights of each criterion, and a consistency check ensures the responses’ logical coherence. The weighted criteria are combined to generate a ranked list of options.

A pairwise comparison matrix was constructed and used to calculate the priority weights for each criterion. The consistency ratio (CR) was calculated to evaluate the consistency of the comparisons. CR values ≤0.10 indicate acceptable consistency, ensuring reliable weight estimation for the AHP model.

### Normalization and sensitivity analysis

Data for each risk factor were normalized on a 0–100 scale. This normalization was performed by multiplying each value by the maximum observed value for the given factor, then dividing by 100. Thus, the continuous variables were rescaled to a comparable range, whereas the categorical variables were converted into numerical scores reflecting their relative contribution to the risk, as determined by expert judgment. Normalized factors were then weighted according to the AHP analysis values. To ensure proportional contributions, all weights were standardized to sum to 1 (100%). The final risk map was produced using a weighted linear combination as follows:

Final map risk = ∑ [Weight (i) × Factor (i)]

Where weight (i) represents the weight assigned to each risk factor and factor (i) represents the normalized risk factor value.

To evaluate the robustness of our model, a sensitivity analysis was performed following several steps. First, the weight of each risk factor was varied individually by ±25%. This amplitude has been widely applied in previous MCDA studies as a reasonable threshold for assessing model stability without introducing unrealistic weight variations. For each modified weight (Pm), the weights of all other factors were re-adjusted to ensure that the total sum remained equal to 1, according to the following formulas:

1. Pm: the weight of the modified factor (increased or decreased by 25%).
2. Pm0: the initial weight of the modified factor.
3. Pi: the normalized weight of each of the other factors.
4. Pi0: the initial weight of each of the other factors (which need to be normalized).

This procedure generated alternative weighting schemes for the introduction and spread models. Each set of weights was then incorporated into the MCDA framework to recalculate the corresponding risk maps.

To quantify the impact of weight variation, 1000 semi-randomly distributed points were generated across the Moroccan territory (using the national boundary as the sampling extent). Risk scores were extracted from the initial map and the modified maps for each sampling point. The change in risk score was then calculated as follows:

∆Risk = Risk modified – Risk initial

Finally, the mean and standard deviation of these variations were calculated for each modified map. This procedure allowed us to identify the most influential risk factors and assess the model’s overall stability under different weighting scenarios.

This sensitivity analysis allowed us to assess the stability of our model results under different weighting scenarios and identify the risk factors that had the most significant influence on the final risk estimates.

### Geospatial analysis platform

In this study, we used an open-source GIS solution, QGIS (version 3.28; https://qgis.org). This enabled us to perform the spatial interpolation of risk variables and route network analysis. Raw risk scores were classified in QGIS using the natural breaks (Jenks) method, where “high risk” corresponds to the highest class determined by the calculated thresholds. This algorithm automatically determines class boundaries by minimizing within-class variance and maximizing between-class variance, thereby identifying the most meaningful groupings in the data distribution. In our case, the number of classes was defined a priori (five), and the Jenks algorithm was applied in QGIS to compute thresholds and assign each spatial unit to its corresponding risk category. This ensured that the risk classes were objectively derived from the data rather than being based on arbitrary intervals.

## RESULTS

### Map of risk factors

In this section, we developed individual risk maps for each risk factor and produced a composite map of the introduction and spread of HPAI in Morocco. The process involved compiling a list of relevant risk factors based on the Moroccan context and data availability, collecting and preparing the geographic database, converting data to raster format, consulting experts to gather opinions, calculating factor weights, combining data layers to generate the final maps, and performing a sensitivity analysis using the mean and standard deviation of the variations in each map.

### Introduction to risk factors

#### Wetland sites

**Figure 1:**
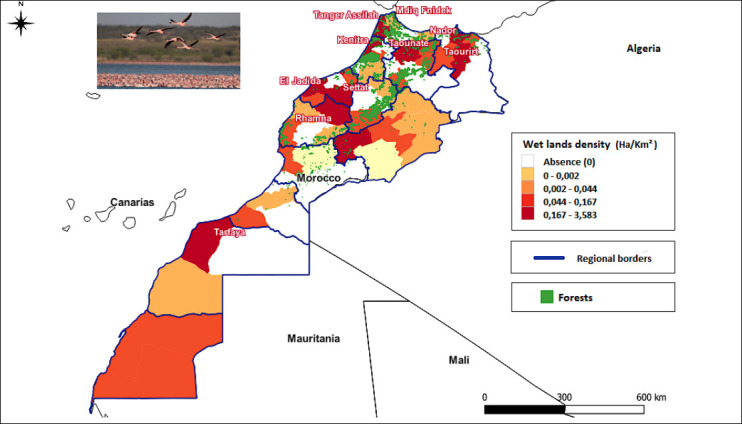
Wetland density in Morocco.

The distribution of wetland density across Morocco revealed distinct risk zones for the introduction of HPAI, as illustrated in Figure 1. High-risk zones, characterized by elevated wetland densities (0.167–3.583 Ha/km2), are concentrated in the northern regions (e.g., Kenitra, Tanger, Assilah, Taounate, and Taourirt), the central coastal area near El Jadida, and the Southwestern region around Tarfaya, key hotspots due to migratory bird pathways. Moderate-risk zones (0.044–0.167 Ha/km^2^) span the extreme south, central-eastern regions, and scattered northern areas, where lower but still significant wetland densities persist. These spatial patterns underscore the critical role of wetlands in avian influenza transmission, underscoring the need for targeted surveillance in high-density regions, particularly along migratory corridors.

#### Importation of live poultry and poultry products

**Figure 2:**
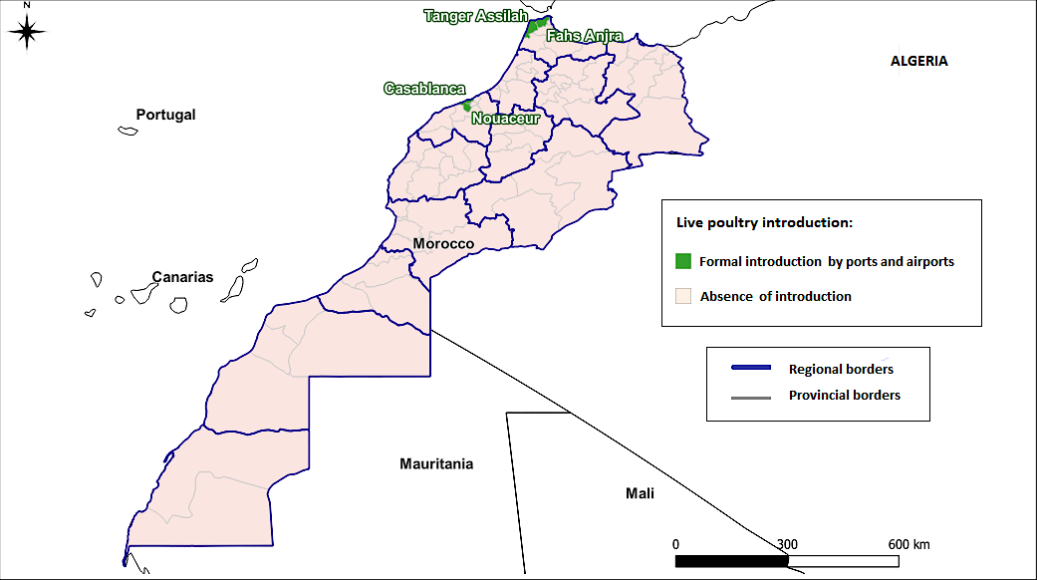
Risk map of highly pathogenic avian influenza through the formal introduction of live poultry in Morocco.

The high-traffic ports and airports of Casablanca and Tangier, which serve as gateways for potential viral entry, are the primary entry points. These hubs directly expose neighboring provinces to heightened risk, including Fahs-Anjra, M’diq-Fnideq Prefecture, Tanger-Assilah Prefecture, and Casablanca-Settat, where imported poultry may enter local markets or production systems. Figure 2 presents Morocco’s formal import routes for live poultry and derived products.

#### Importation of recreational birds

**Figure 3:**
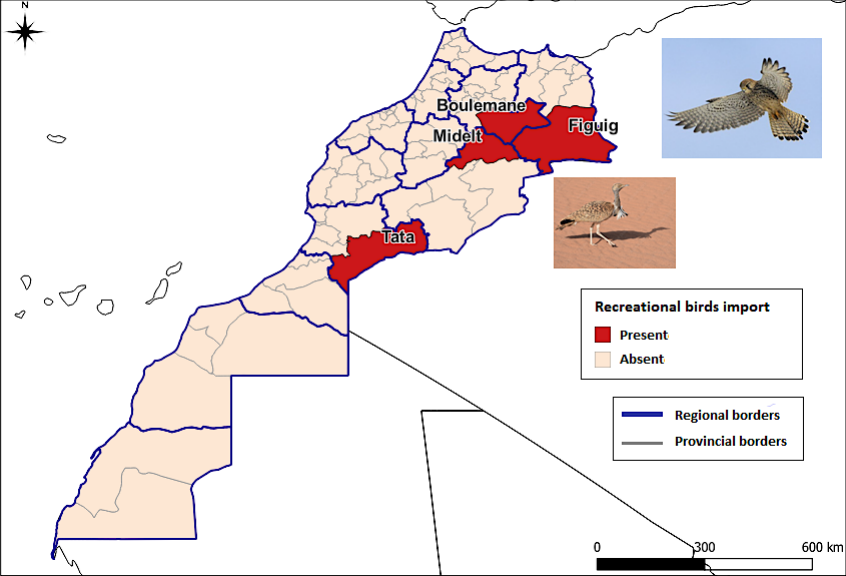
Map of the risk of introduction of highly pathogenic avian influenza through recreational bird imports.

The importation of recreational birds in certain areas of Morocco suggests a potential pathway for the introduction of HPAI. Key locations, such as Boulemane, Midelt, Tata, and Figuig, represent zones where this activity occurs. Figure 3 illustrates the distribution of this activity, highlighting specific regions where it is present or absent.

### Spread risk factors

#### Biosecurity levels, density, and type of productions

Based on expert consultations, a biosecurity score was assigned to each production type. Table 3 presents the experts’ scores for each farm type, reflecting their relative level of biosecurity implementation. The highest average biosecurity score (4.08) was attributed to broiler chicken farms, reflecting a potential risk for the spread of HPAI. In contrast, hatcheries and breeder farms had the lowest scores (1.92 and 2.00, respectively), indicating relatively better biosecurity measures and lower transmission risks.

**Table 3:** The score attributed to each type of farm according to the level of biosecurity.

Type of production	Very low	Low	Medium	High	Very high	Score
Broiler turkey	4	7	21	4	3	3.13
Broiler chicken	17	10	11	0	1	4.08
Laying hens	0	4	9	18	8	2.23
Breeder	0	4	7	13	15	2.00
Hatcheries	0	4	6	12	17	1.92

**Figure 4:**
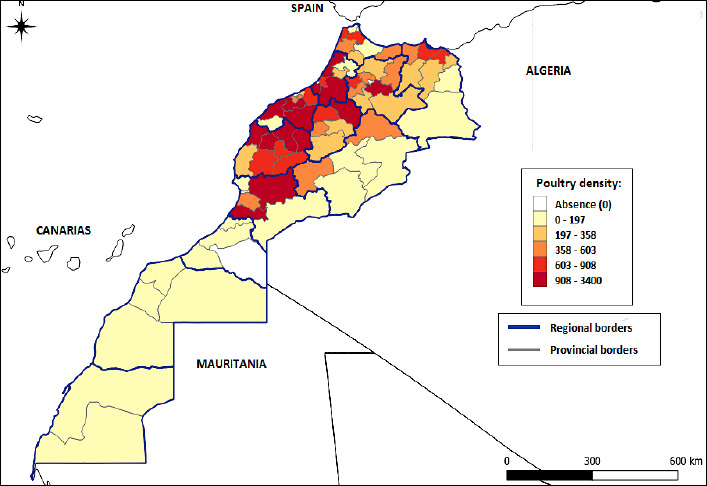
Risk map of highly pathogenic avian influenza spread according to poultry farm density, production type, and biosecurity levels in Morocco.

The final risk score for each province, calculated by integrating the number of poultry units, farm type, and associated biosecurity levels is shown in Figure 4. These data reveal regional disparities in the transmission risk of HPAI. Critical hotspots are present in west-north-central regions where high-density operations (908–3,400 units) coincide with inadequate biosecurity. These areas emerge as intervention priority zones.

#### Live poultry and table egg markets

The concentration of the poultry trade through Morocco’s wholesale markets poses a significant risk for the spread of HPAI. Current data indicate that approximately 50% of table eggs and over 75% of slaughter-bound poultry are channeled through two major wholesale markets in Casablanca and Rabat [45]. This extreme centralization creates critical biosecurity vulnerabilities, as these hubs serve as primary mixing points for birds from multiple production systems with varying biosecurity standards (from industrial farms to backyard operations), high-traffic nodes where pathogens can amplify, and distribution centers that could rapidly disseminate infections nationwide [37, 46]. The proximity of these markets to Morocco’s high-density poultry production zones further compounds the transmission risks.

#### Movement of means of transport linked to the poultry sector

**Figure 5:**
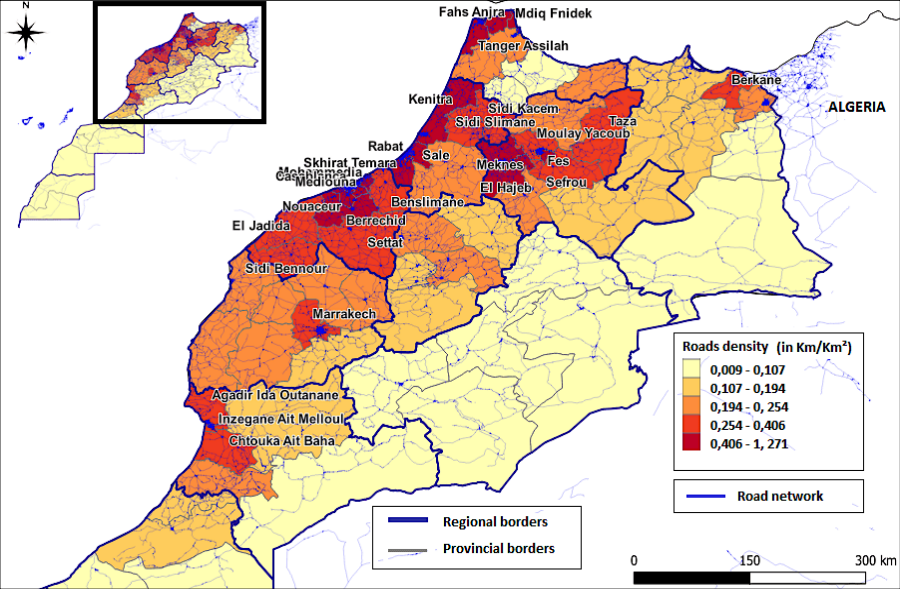
Risk map of highly pathogenic avian influenza spread according to road density in Morocco.

The generated map (Figure 5) illustrates the risk of HPAI spread in Morocco based on road density (measured in km/km^2^). Using a color gradient from pale yellow (lowest density: 0.009–0.107) to dark red (highest density: 0.406–1.271), the map highlights that major urban centers, such as Rabat, Casablanca, Marrakech, and Tangier, have the highest road densities, making them potential hotspots for disease transmission.

The Atlantic coastal regions have higher road connectivity than the eastern and southeastern areas bordering Algeria, which have minimal road infrastructure. This visualization effectively demonstrates how transportation networks could facilitate the spread of HPAI through poultry movement and human activity, with risk levels directly corresponding to the density of road networks across Morocco’s provinces and regions.

#### Density of the human population

**Figure 6:**
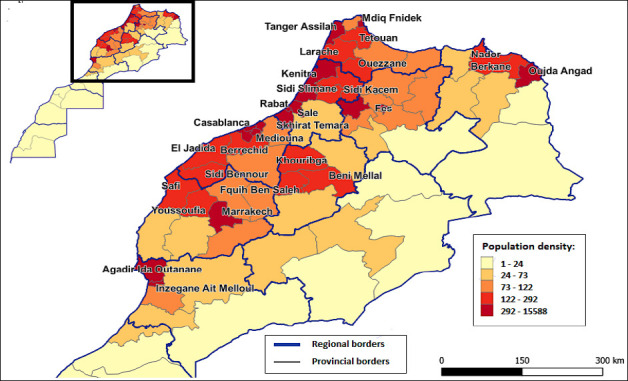
Risk map of highly pathogenic avian influenza spread according to the human population density in Morocco.

This map (Figure 6) illustrates the spatial distribution of human population density across the Moroccan provinces. The highest densities were concentrated in major urban and peri-urban areas such as Casablanca, Rabat-Salé, Marrakech, Fès, and the northern axis Tanger-Tétouan, where values exceed 292 inhabitants/km2 and may reach more than 15,000 in some urban districts. Intermediate densities (73–292 inhabitants/km2) are observed in provinces combining medium-sized cities and rural surroundings, whereas low densities (<73 inhabitants/km2) dominate rural, mountainous, and southern provinces. From an epidemiological perspective, high-density zones represent areas of greater vulnerability to the spread of HPAI H5N1, as human concentration facilitates virus circulation through trade and agricultural practices.

### Calculated risk factor weights

Each expert’s opinion was then processed to determine the weights of each factor. After processing the questionnaire and analyzing the expert’s opinions, we obtained the weights of the risk factors of the introduction and spread (Tables 4 and 5).

**Table 4:** The weight of risk factors for highly pathogenic avian influenza introduction assigned by experts.

Risk factor	Wetland sites	Importation of live poultry	Importation of recreational birds	Importation of poultry products
Weights	0.4405	0.1776	0.2499	0.1320

**Table 5:** Weight of highly pathogenic avian influenza spread risk factors assigned by experts.

Risk factor	Density and type of poultry production	Live poultry and table eggs markets	Movement of means of transport linked to the poultry sector	Population density
Weights	0.2644	0.2666	0.3359	0.1332

### MCDA map editing

**Figure 7:**
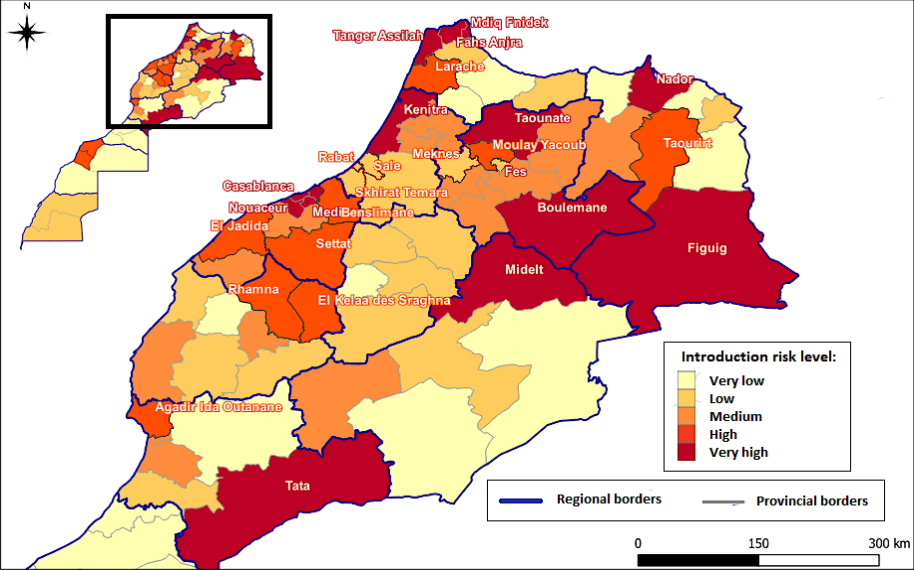
Map of the risk of introduction of the highly pathogenic avian influenza virus in Morocco.

**Figure 8:**
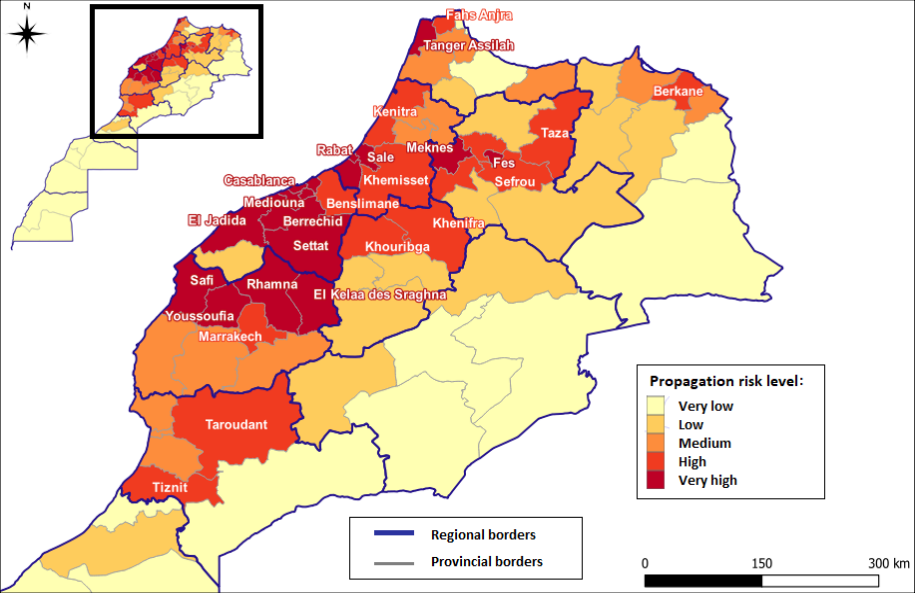
Map showing the spread risk of the highly pathogenic avian influenza virus in Morocco.

Maps of the HPAI introduction risk and spread have been prepared based on the full MCDA process (Figures 7 and 8), providing a comprehensive risk assessment of HPAI in Morocco.

The first map (Figure 7) depicts the spatial distribution of the introduction risk of the HPAI virus across the provinces of Morocco based on the MCDA method. Risk is expressed in five classes, ranging from very low (light yellow) to very high (dark red). Areas shown in dark red (very high risk) include Tangier-Assilah, Fahs Anjra, Boulemane, Figuig, Midelt, and Tata. These high-risk zones are primarily concentrated in the northern coastal regions (particularly around Tangier), characterized by dense poultry farming and proximity to migratory bird flyways; the eastern border areas with Algeria, where cross-border poultry movement occurs; and some southern regions. Moderate-to-high introduction risk is observed in urban and peri-urban areas, such as Rabat, Casablanca, and their surrounding provinces, reflecting high poultry density and market activity.

The second map (Figure 8) illustrates the spread risk of HPAI once it is introduced. The highest risk of spread (dark red) is concentrated along the western coastal corridor, particularly in Safi, Berrechid, Settat, Benslimane, Mediouna, and Casablanca. This pattern corresponds with areas of high poultry production, dense human population, and major transportation routes that facilitate rapid disease dissemination.

In summary, Table 6 presents the Moroccan provinces identified as being at very high risk of both the introduction and spread of HPAI.

**Table 6:** Very high risk of highly pathogenic avian influenza introduction and spread in Morocco and the provinces of concern.

Type of risk	Provinces concerned
Very high risk of introduction	Fahs Anjra, Tanger Assilah, M’diq Fnidek, Nador, Kenitra, Taounate, Fès, Midelt, Boulemane, Figuig, Casablanca, Tata, Médiouna–Nouacer, and Mohammedia
Very high risk of spread	Casablanca, Rabat, Fès, El Jadida, Settat, Mohammedia, El Kelaa des Sraghna – Rhamna, Safi – Youssoufia, Médiouna – Nouacer, Salé, Berrechid, Skhirat – Témara, Tanger Assilah, Meknès

### Sensitivity and uncertainty analysis

The results showed that increasing or decreasing the weight of individual risk factors resulted in negligible changes in the risk scores. The highest average variation in the probability score was 1.62 ± 5.51, due to the increased weight attributed to the density and production type of poultry farms in the spread of the disease. In other words, changing the weight attributed to the density and type of production of poultry farms by 25% should only change the final overall risk score for the spread of avian influenza by −3–7 points on a scale of 100. This suggests that the developed MCDA model is robust.

## DISCUSSION

### Risk of introduction of HPAI

The risk of avian influenza introduction in Morocco is at its highest level across seven of 12 administrative regions. This risk can be associated with three probable origins: (i) Points of entry of live poultry (day-old chicks and recreational birds) through ports and airports, (ii) release of captive-bred prey for falconry, and (iii) the presence of large wetlands frequented by migratory birds.

#### Entry points of live poultry

Ports and airports represent critical points of entry for avian influenza. This risk particularly affects the provinces of Tangiers-Assilah, Fahs-Anjra, Casablanca, and Nouacer. However, this pathway is controlled through monitoring and control programs implemented by the Moroccan veterinary authority (ONSSA) at points of entry.

#### Recreational bird importation

Another important origin of risk is the release of captive-bred prey in connection with falcon hunting, notably through activities associated with the Emirates Center for Wildlife Propagation [32]. This concerns provinces such as Figuig, Boulemane, Midelt, and Tata. Importation of recreational birds, particularly falcons, is a significant factor in the epidemiology of avian influenza in Morocco. Previous studies [10–12, 47] have demonstrated a strong genetic similarity between LPAI strains circulating in Morocco and those from the Middle East, suggesting virus introduction through falcon movement, a concern given the prevalence of recreational hunting activities in certain provinces.

#### Wetlands and migratory bird corridors

Large wetlands, particularly in Morocco’s northwestern and Mediterranean regions, constitute one of the most subtle and difficult-to-control sources of avian influenza introduction. These habitats, situated along migratory flyways, are key stopover points for diverse bird species that may carry avian influenza virus asymptomatically [48, 49].

Key ecological sites include:

1. Province of Kenitra: Sidi Boughaba Lake (6 km length; 205 species, including 177 migratory) [50] and Merja Zerga Lagoon (8–9 km long, 5 km wide; over 100 species; critical East-Atlantic flyway site) [51, 52].
2. Province of Mdiq-Fnidek: Smir Lagoon and Dam (837 ha; more than 100 migratory bird species) [53].
3. Province of Taounate: My Driss Ier Dam wetland, a wintering site for over 40 migratory species [53].
4. Mediterranean Coastline: Nador Lagoon (15,000 ha; over 120 migratory and breeding bird species; high ecological interest) [54].

These wetlands are favorable gathering sites for large bird concentrations, especially in autumn and winter, with risks peaking in February and lowest in September [55].

A second stratum of risk extends inland and to southern Morocco, including:

1. El Massira Dam (Settat Province): >50 wintering migratory species [50].
2. Sidi Moussa El Walidia (El Jadida Province): >114 waterbird species [56].
3. Mohammed V Dam (Taourirt Province): Up to 45,000 waterbirds annually [50].
4. Oued Souss (Agadir Province) and Khenifiss National Park (Tarfaya Province): >179 species; >20,000 waterbirds annually [53].

### Risk of spread of HPAI

#### Poultry density and biosecurity

The highest risk of spread is associated with provinces that have dense poultry farms, particularly broiler and turkey operations, where biosecurity measures are weaker compared to layer and breeder farms [37]. Numerous studies confirm that poultry unit density correlates positively with HPAI contamination risk [57] and inadequate biosecurity significantly amplifies pathogen spread [58].

#### Transport networks and human population density

High road density also facilitates viral dissemination during epizootics. Provinces with large human populations are major consumption centers, often with insufficient hygiene and biosecurity in traditional poultry markets and slaughter channels.

This combined risk is particularly high in:

1. Casa–Settat region and the northern half of the Marrakech–Safi region, which produce >25% of national poultry.
2. Skhirate-Temara, Rabat, and Salé (Rabat–Salé–Kenitra Region).
3. Fès and Meknes (Fès–Meknes Region).
4. Tangier-Assilah (northern Morocco).

#### Model validation and expert opinion

Model validation revealed values of 0.61 (introduction) and 0.70 (spread). These values are lower than expected, largely due to reliance on LPAI H9N2 outbreak data from 2016, since Morocco has not yet experienced HPAI. That outbreak involved only 61 farms (out of ~10,000) and had a daily mortality rate of ~2.67% [10]. Early national control measures, awareness campaigns, strengthened biosecurity, and preventive vaccination, limited the spread [37, 58].

Variations in expert opinions were also noted, influenced by their professional backgrounds (professors, researchers, private or government veterinarians). Such subjectivity is a known limitation of MCDA [14, 16]. However, sensitivity analysis confirmed robustness: Altering individual factor weights by ±25% had no significant impact, consistent with previous findings in Africa and Indonesia [59]. Moreover, the number of experts consulted exceeded sample sizes in comparable studies [60], improving reliability.

### Recommendations

To reduce risks, preventive and control measures should be prioritized in provinces with the highest combined risk, namely:

1. Casablanca–Settat and northern Marrakech–Safi
2. Skhirate–Temara, Rabat, Salé
3. Fès and Meknes
4. Tangier-Assilah.

- Key recommendations include:

1. Active surveillance in high-risk provinces.
2. Enhanced biosecurity at live bird markets and poultry farms.
3. Efficient allocation of vaccines, diagnostics, and trained personnel.

Finally, the MCDA method proved advantageous due to its ease of use and transferability to field stakeholders. The risk maps developed here provide a practical decision-support tool, guiding policymakers and supporting the design of risk-based surveillance and control programs.

## CONCLUSION

This study represents the first comprehensive attempt to map the spatial risk of HPAI introduction and spread in Morocco using an MCDA integrated with GIS. The results indicate that seven of the 12 administrative regions face the highest risk of introduction, primarily driven by three pathways: The entry of live poultry and recreational birds through ports and airports (Tangiers-Assilah, Fahs-Anjra, Casablanca, Nouacer); the release of captive-bred prey linked to falconry in provinces such as Figuig, Boulemane, Midelt, and Tata; and the extensive wetland ecosystems along Morocco’s northwestern and Mediterranean flyways, including Merja Zerga, Sidi Boughaba, and Nador Lagoon, which host large concentrations of migratory birds. Spread risk was highest in provinces with dense poultry production, inadequate biosecurity in broiler and turkey farms, extensive transport networks, and large human populations, particularly in the Casablanca–Settat and northern Marrakech–Safi regions, as well as Skhirate-Temara, Rabat, Salé, Fès, Meknes, and Tangier-Assilah.

These findings highlight the need for risk-based interventions. Prioritizing active surveillance, strengthening biosecurity at farms and live bird markets, and allocating vaccines, diagnostics, and trained personnel to high-risk provinces will significantly reduce the likelihood of HPAI introduction and its subsequent dissemination. The generated risk maps provide Moroccan veterinary authorities with a decision-support tool to guide early detection, resource allocation, and tailored prevention strategies.

The main strengths of this study lie in the integration of expert knowledge with spatial data, enabling risk prediction in a country without reported HPAI outbreaks. The use of MCDA combined with ROC-based validation, sensitivity analysis, and multiple data layers ensured a robust methodological framework adaptable to Morocco’s specific epidemiological and ecological context. However, limitations must be acknowledged. Validation relied on LPAI H9N2 outbreak data from 2016 due to the absence of HPAI cases in Morocco. Expert-based weighting introduces subjectivity, though this was mitigated by involving a diverse group of 73 professionals. In addition, surveillance data gaps may underestimate risks in regions with limited monitoring capacity.

Future research should refine the model by incorporating real-time surveillance data, genetic sequencing of circulating strains, and socio-economic drivers such as informal poultry trade networks. Cross-border data sharing with neighboring countries and integration of satellite-tracked migratory bird movements could further enhance predictive accuracy. Expanding the framework to regional applications in North Africa and the Mediterranean would strengthen collective preparedness against transboundary avian influenza threats.

In conclusion, Morocco faces tangible risks of HPAI introduction and spread, particularly in provinces with high poultry density, trade connectivity, and ecological exposure to migratory birds. While Morocco has so far been spared from HPAI incursions, its endemic LPAI circulation, proximity to affected countries, and role along major flyways underscore the urgency of adopting proactive, risk-based surveillance and control measures. The MCDA approach used here offers a practical and adaptable framework for national authorities, ensuring preparedness and resilience against potential future outbreaks.

## DATA AVAILABILITY

This study is part of an ongoing research project funded by the European Union (WiLiMan ID). Part of the data will be used in follow-up studies that are currently in progress. These data can be accessed through Contact person: Fellahi Siham, Institut Agronomique et Vétérinaire Hassan II. Email: s.fellahi@iav.ac.ma. We commit to making these data available to qualified researchers who meet the criteria for access to confidential data, subject to approval from our institutional review board and funding body.

## AUTHORS’ CONTRIBUTIONS

FB, SF, OA, KA, MD, and HH: Conceptualized the study. FB, SF, KA, and HH: Curated the data and performed the formal analysis. FB: Drafted the original manuscript. FB, SF, OA, KA, HH, and MD: Reviewed and edited the manuscript. All authors have read and approved the final version of the manuscript.
